# Mobility Skills With Exoskeletal-Assisted Walking in Persons With SCI: Results From a Three Center Randomized Clinical Trial

**DOI:** 10.3389/frobt.2020.00093

**Published:** 2020-08-04

**Authors:** EunKyoung Hong, Peter H. Gorman, Gail F. Forrest, Pierre K. Asselin, Steven Knezevic, William Scott, Sandra Buffy Wojciehowski, Stephen Kornfeld, Ann M. Spungen

**Affiliations:** ^1^Spinal Cord Damage Research Center, James J. Peters VA Medical Center, Bronx, NY, United States; ^2^Department of Rehabilitation and Human Performance, Icahn School of Medicine at Mount Sinai, New York, NY, United States; ^3^Department of Neurology, University of Maryland School of Medicine and Chief, Division of Rehabilitation Medicine, University of Maryland Rehabilitation and Orthopaedic Institute, Baltimore, MD, United States; ^4^Center for Spinal Stimulation and Center for Mobility and Rehabilitation Engineering, Kessler Foundation, West Orange, NJ, United States; ^5^Physical Medicine & Rehabilitation, Rutgers New Jersey Medical School, Newark, NJ, United States; ^6^Department of Neurology, University of Maryland School of Medicine and VA Maryland Healthcare System, Baltimore, MD, United States; ^7^Performance Exercise Attitude and Knowledge Center, Craig Hospital, Englewood, CO, United States; ^8^Department of Medicine, Icahn School of Medicine at Mount Sinai, New York, NY, United States

**Keywords:** exoskeletal-assisted walking, mobility walking tests, 10MWT, 6MWT, TUG, Food and Drug Administration

## Abstract

**Background:** Clinical exoskeletal-assisted walking (EAW) programs for individuals with spinal cord injury (SCI) have been established, but many unknown variables remain. These include addressing staffing needs, determining the number of sessions needed to achieve a successful walking velocity milestone for ambulation, distinguishing potential achievement goals according to level of injury, and deciding the number of sessions participants need to perform in order to meet the Food and Drug Administration (FDA) criteria for personal use prescription in the home and community. The primary aim of this study was to determine the number of sessions necessary to achieve adequate EAW skills and velocity milestones, and the percentage of participants able to achieve these skills by 12 sessions and to determine the skill progression over the course of 36 sessions.

**Methods:** A randomized clinical trial (RCT) was conducted across three sites, in persons with chronic (≥6 months) non-ambulatory SCI. Eligible participants were randomized (within site) to either the EAW arm first (Group 1), three times per week for 36 sessions, striving to be completed in 12 weeks or the usual activity arm (UA) first (Group 2), followed by a crossover to the other arm for both groups. The 10-meter walk test seconds (s) (10MWT), 6-min walk test meters (m) (6MWT), and the Timed-Up-and-Go (s) (TUG) were performed at 12, 24, and 36 sessions. To test walking performance in the exoskeletal devices, nominal velocities and distance milestones were chosen prior to study initiation, and were used for the 10MWT (≤ 40s), 6MWT (≥80m), and TUG (≤ 90s). All walking tests were performed with the exoskeletons.

**Results:** A total of 50 participants completed 36 sessions of EAW training. At 12 sessions, 31 (62%), 35 (70%), and 36 (72%) participants achieved the 10MWT, 6MWT, and TUG milestones, respectively. By 36 sessions, 40 (80%), 41 (82%), and 42 (84%) achieved the 10MWT, 6MWT, and TUG criteria, respectively.

**Conclusions:** It is feasible to train chronic non-ambulatory individuals with SCI in performance of EAW sufficiently to achieve reasonable mobility skill outcome milestones.

## Introduction

Paralysis resulting from spinal cord injury (SCI) often leads to a reduction in mobility and an associated decrease in daily physical activity. In addition, SCI also leads to other secondary adverse consequences related to body composition (Wilmet et al., 1995; Spungen et al., 1999, 2000), cardiovascular function (Wahman et al., 2010; LaVela et al., 2012), autonomic integrity (Wecht et al., 2000, 2001), and bowel function (Glickman and Kamm, 1996; Stiens et al., 1997; Korsten et al., 2004). The combination of reduced mobility and secondary consequences of SCI leads to a reduced quality of life (Costa et al., 2001; Tate et al., 2002; Jensen et al., 2007; Wilson et al., 2011; Munce et al., 2013).

Devices classified by the Food and Drug Administration (FDA) as “powered exoskeletons” (Product Classification U. S. Food Drug Administration, 2019) have become commercially available and enable individuals with motor paralysis to stand and walk over ground. These devices employ use of a ridged external frame for bracing the lower extremities and trunk. Rechargeable battery powered motors are then used to power movement of the hip and knee joints. Just as able bodied walking requires the ability to maintain balance and perform weight shifting (Tapio, 2016), powered exoskeleton assisted ambulation requires the same. These movements are measured by sensors in the device that trigger motors to power movement at the hip and knee joints. Consecutive weight shifting must be completed by actively maintaining balance on the stance leg so that the swing leg can clear the floor appropriately. Subsequent weight shift onto the contralateral side continues to trigger the device to take steps. Over ground balance maintenance and weight shifting are assisted through use of crutches or a walker. The execution of this exoskeletal-assisted walking (EAW) movement places demands on the neuromuscular and sensory systems of the user, increasing oxygen consumption when compared to able bodied ambulation (Asselin et al., 2015; Evans et al., 2015). The additional metabolic activity required to ambulate with these devices has the potential to provide a cardiovascular exercise challenge and thereby improve cardiovascular health (Escalona et al., 2018). However, since this technology remains relatively new, many aspects of its use by persons with SCI have yet to be determined.

Due to limitations with current available systems, not all persons with SCI are able to successfully achieve EAW (walking velocity of ≥0.40 m/s over 10 meters and 6-min walk distance ≥110 m with minimal assistance or less). Some users may manage to take steps but require a significant amount of assistance to accomplish this. Therefore, identification of basic skills during early sessions in order to predict who would be potential responders, that is successful and independent users of the device in the home and community after completing a training program, would be important. The purpose of this study was to document the number of sessions necessary to achieve adequate EAW skills and velocity milestones, to document the proportion of participants who achieved successful EAW skills by 12 sessions, and to determine the skill progression over the course of 36 sessions.

## Method

### Recruitment

This study was approved by the Institutional Review Boards (IRB) of the three collaborating clinical sites, namely the James J. Peters VA Medical Center (JJPVAMC), Bronx, NY, Kessler Institute for Rehabilitation/Kessler Foundation (KIR/KF), West Orange, NJ, and the University of Maryland, Baltimore IRB for the University of Maryland Rehabilitation and Orthopedic Institute (UM Rehab and Ortho), Baltimore, MD). In addition, the Department of Defense Congressionally Directed Medical Research Program (DOD CDMRP) IRB approved the total study. Several recruitment strategies were employed. The study physicians at each site were the primary source of identifying potential participants. In-services at each site were provided to educate other staff physicians about this study for referrals. Additionally, at each site IRB-approved flyers and brochures were distributed. Physician-referred potential participants, as well as those responding to IRB approved advertisements or the clinicaltrials.gov website listing (NCT02314221), were informed about the details and eligibility for the study. The targeted study population included persons with chronic SCI (≥6 months) who were non-ambulatory and therefore used wheelchairs for mobility. The inclusion/exclusion criteria of this study are described below (Table 1).

**Table 1:** Enrollment criteria.

**Enrollment criteria**
*Inclusion criteria:* 1. Males and females, between 18 and 65 years old; 2. Traumatic or non-traumatic tetraplegia or paraplegia >6 months in duration; 3. Unable to ambulate faster than 0.17 m/s on level ground with or without an assistive device and are wheelchair-dependent for mobility; 4. Height 160 to 190 cm (63–75 in or 5'3” to 6'3” ft) 5. Weight <100 kg (<220 lb) 6. Able to hold the Lofstrand crutches or wheeled walker; and 7. Able to sign informed consent.
*Exclusion criteria:* 1. Diagnosis of neurological injury other than SCI including: a Multiple sclerosis, Stroke, Cerebral Palsy, Amyotrophic lateral sclerosis, Traumatic Brain injury, Spina bifida, Parkinson's disease, or b Other neurological condition that the study physician considers in his/her clinical judgment to be exclusionary; 2. Severe concurrent medical disease, illness or condition; 3. Lower extremity fracture within the past 2 years; 4. Dual Energy X-ray Absorptiometry (DXA) results indicating a t-score below−3.5 at the femoral neck or the total proximal femur bone and knee bone mineral density (BMD) <0.60 gm/cm2; 5. Diagnosis of heterotopic ossification of the lower extremities which affect range of motion or proper BMD measurements; 6. Significant contractures defined as flexion contracture limited to 35° at the hip and 20° at the knee; 7. Untreated hypertension (SBP>140, DBP>90 mmHg); 8. Symptomatic orthostatic hypotension during standing that does not resolve after attempts at upright posture that were made over several days, and standing by the participant is deemed to pose a health risk, as determined by a physician, because of symptomatic orthostatic hypotension; 9. Systemic or peripheral infection; 10. A medical diagnosis in the patient chart of atherosclerosis, congestive heart failure, or history of myocardial infarction; 11. Trunk and/or lower extremity pressure ulcers; 12. Severe spasticity (defined by an Ashworth score of 4.0 across a lower extremity joint or clinical impression of the study physician or physical therapist); 13. Significant contractures defined as flexion contracture limited to 25° at the hip and knee; 14. Diagnosis of heterotopic ossification of the lower extremities which affect range of motion or proper measurement of BMD measurements; 15. Psychopathology documentation in the medical record or history of that may conflict with study objectives; 16. Pregnancy and/or lactating females. 17. Brain injury with score on mini-mental status examination <26 18. Diagnosis of coronary artery disease that precludes moderate to intense exercise; 19. Deep vein thromboses in lower extremities of <6 months duration; 20. Other illness that the study physician considers in his/her clinical judgment to be exclusionary.

### Protocol

Participants were screened for eligibility after signing the informed consent form. Screening tests for eligibility included a complete history and physical examination incorporating the following: the International Standards for Neurological Classification of SCI (ISNCSCI) examination to determine level and completeness of injury, range of motion at the hips, knees and ankles bilaterally, Ashworth spasticity examination in the lower extremities, standing orthostatic tolerance test, and bone mineral density (BMD) scanning of bilateral knees (proximal tibia and distal femur) and hips (femoral neck and total hip) by Dual Energy X-ray Absorptiometry (DXA). Exclusion criteria for the BMD measurements have been described (Table 1).

Eligible participants were randomized within site to one of two groups for 12 weeks (3 months): Group 1 received EAW first for 12 weeks then crossover to usual activity (UA) for a second 12 weeks; Group 2 received UA first for 12 weeks then crossover to EAW for 12 weeks of training. The EAW arm consisted of EAW training, three sessions per week (4–6 h/week) for 36 sessions. The UA arm consisted of identification of usual activities for each participant and encouragement to continue with these activities throughout the 12-week UA arm. This study employed a randomized, crossover design with an EAW intervention arm and an UA arm which was designed to serve as a control arm for the secondary outcomes of the clinical/medical variables. The results of the medical variables are beyond the scope of the present manuscript and will be presented in a future work. As such, the UA arm was not intended to be used as a control comparison for the mobility outcome measures.

**Figure 1:**
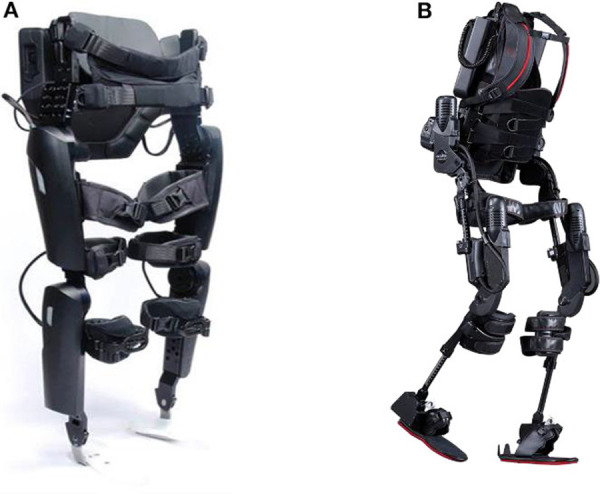
Exoskeletons used in this study: **(A)** ReWalk (ReWalk Robotics, Inc. Marlborough, MA, USA) and **(B)** Ekso GT (Ekso Bionics, Richmond, CA, USA).

Two powered exoskeleton devices (Figure 1) were used in this study, namely the ReWalk™ (ReWalk Robotics, Marlborough, MA)^1^ and the Ekso™ (Ekso Bionics, Richmond, CA)^2^. These powered exoskeletons were chosen because they were the only devices commercially available and FDA approved for use within rehabilitation centers at the time of study development. In addition, the ReWalk has been FDA approved for home and community use based on certain user characteristics and achievements within a supervised rehabilitation center (spinal injury level T7 to L5, walking velocity of ≥0.4 m/s over 10 meters and 6 min walk distance ≥110 m) (Hoffmann, 2016). Both devices have functional similarities, such as the required concurrent use of Lofstrand crutches or a wheeled walker in the case of the Ekso, and the need for the user to shift their weight in order to trigger sensors that in turn motorized the hip. However, there are some notable differences in the specifications of the devices such as the stepping pattern and the design such as the footplate in the Ekso. The maximum documented velocities for the devices are 0.80 m/s for the ReWalk^1^ and 0.45 m/s for the Ekso^2^. The Ekso device has a rigid back that provides thoracic support to accommodate participants who have less trunk stability such as those with a low cervical (C) or high thoracic (T) level of injury. The footplate of Ekso has a sensor to detect weight shifts and assist with triggering the hip motors. The foot trajectory of the Ekso follows a semi-elliptical trajectory with a higher step height for foot clearance, leading to a marching style gait pattern. In the Ekso, depending on the functional abilities of the user, the level of device assist could be selected from the adaptive, maximal or fixed mode. In response to the participants' functional abilities, variable assistance constantly adapts motor output or a fixed level of assistance could be set for participants. The ReWalk gait paradigm is more of a swing pattern minimizing the step height but requiring more controlled balance to successfully achieve reciprocal stepping (Asselin et al., 2016). Thus, the ReWalk powered exoskeleton was chosen primarily for participants with injury levels at T3 and below who could perform weight shifting and clear each foot during stepping. Those with higher cord lesions and less trunk stability were better able to utilize the Ekso powered exoskeleton. Although choice of which device was commonly distributed based on level and completeness of injury, device selection was somewhat variable depending on the participant's preference and the clinical judgment of the study team. Both devices were used at JJPVAMC and Kessler, and the ReWalk was only used at UM Rehab and Ortho. Study-related serious adverse event (SAE) and adverse event (AE) tracking occurred throughout the study.

### Training Sessions

Generally, within the first two sessions, standing balance skills were practiced and achieved prior to progression to walking skills. Walking skills began with unloading the right foot (both devices use the right leg to take the first step). Shifting weight onto the right foot and unweighing the left foot was the next step in the progression of walking. Continuous walking resulted from serial performance of the anterior-lateral diagonal shift onto the contralateral limb. Because this was an entirely new skill for the participants, mobility outcomes were not measurable at time 0 (baseline). It was important to determine how many participants could achieve successful EAW skills by 12 sessions to prove or disprove clinical relevance and to project progression by 36 sessions. Participants were asked to perform EAW sessions three times/week for 12 weeks. During each session, heart rate (HR), blood pressure (BP), total steps and rating of perceived exertion (RPE, by the Borg scale: from 6 to 20) (Escalona et al., 2018)^3^ were monitored. Additional details of the training program were presented previously (Asselin et al., 2016; Baunsgaard et al., 2018). Missed sessions (due to transportation, weather, etc.) were added on to the length of the training period when possible in order to achieve a total of 36 sessions. A modified Functional Independence Measure (FIM) was used to assess how much physical assistance from the trainer was provided to participants in order to complete mobility skills. The FIM scale (7: Complete Independence, 6: Modified Independence, 5: Supervision, 4: Minimal Assist, 3: Moderate Assist, 2: Maximal Assist, 1: Total Assist, 0: Activity does not occur) (Dodds et al., 1993; FIM, 2019) for level of assistance during EAW was used and ranged from 0 to 6. While a score of 7, complete independence, is a part of the FIM scale, it is not applicable for this study as all participants required the use of the exoskeleton, thus negating the ability of complete independence (Dodds et al., 1993; FIM, 2019).

### Outcome Measures

A variety of walking assessments were employed to assess an individual's functional independence (Shinkai et al., 2000; Middleton et al., 2015). The 10-meter walk test (10MWT), which measures the time in seconds (s) taken to walk 10 meters, is a short distance performance measurement to determine functional mobility and vestibular function (Van Hedel et al., 2008)^4^. The 6 min walk test (6MWT) is a submaximal exercise test that measures the distance in meters (m) traversed over 6 min and provides cardiopulmonary and musculoskeletal functional capacity information (Van Hedel et al., 2008; Bittner and Singh, 2017). The timed-up-and-go (TUG) is the time from the starting in a seated position to stand-up, walk ten feet, turn around, walk back ten feet, and sit down. This measurement was performed to assess fall risk and ability to balance and maneuver the device during the sit-to-stand and stand-to-sit procedures (Podsiadlo and Richardson, 1991; Van Hedel et al., 2008). During all walking tests, level of assistance, balance maintenance, weight shifts, reciprocal stepping and functional mobility were observed and recorded. The three walking test measurements were performed during the 12th, 24th, and 36th sessions.

### Data Analysis

All statistical tests were set *a priori* at alpha = 0.05. Descriptive statistics and frequency distributions were used to describe the demographic data. All statistical analyses were completed using SPSS 23.00 or higher. The continuous variables were reported in mean plus or minus standard deviation. Total steps over 36 sessions and average of steps were calculated to determine participants' overall performance during this study. Because of differences in characteristics of devices, number of steps and velocity were categorized by devices. With each walking outcome (10MWT, 6MWT, and TUG), achievement of the hypothesized goals during the EAW intervention were reported as categorical data and presented as percent occurrence. The hypotheses for significant positive changes at session 12 verses session 36 for the EAW walking tests were as follows: at session 12, 10% of participants would complete the 10MWT in ≤ 40 s and 20% of the participants would complete the 6MWT of ≥80 m and TUG in ≤ 90 s; at session 36, 70% of participants would complete the 10MWT in ≤ 40 s and 6MWT of ≥80 m and 60% of participants would perform the TUG in ≤ 90 s. Additional analyses were performed according to skill level of completing the 10MWT, 6MWT and TUG categorized by slow, medium, and fast velocity sub-groups. The velocity sub-groups were defined *post hoc* after the review of data starting with using the FDA criteria as the minimum velocity for “fast” and thus representing those with the greatest skill level in the devices. The “medium” velocity was defined as those who could walk at speed and distance ranges that demonstrated some proficiency with the devices, and “slow” were those who were minimally able to use the devices. The velocities and distances by category for each walking test are provided (Table 2). To determine significant main effects, the mobility skills were evaluated for the three different time points using a repeated measure analysis of variance (ANOVA). *Post hoc* analysis were performed using paired *t*-tests to determine significance between sessions 12 and 36 for progression of participant performances on the mobility outcomes. Additionally, the TUG criterion was analyzed further and compared to the established FDA criteria for the 10MWT (speed ≥0.40 m/s) and 6MWT (distance ≥110 m).

**Table 2:** Exoskeletal-assisted walking velocity categories for each of the walking tests.

**EAW category**	**10MWT**	**6MWT**	**TUG**
Slow	<0.25 m/s	<80 m	≥120 s
Medium	≥0.25 and <0.40 m/s	≥80 and <110 m	≥90 and <120 s
Fast	≥0.40 m/s	≥110 m	<90 s

*10MWT, 10-minute walk test; 6MWT, six-meter walk test; TUG, timed up and go; m/s, meters per second; m, meters; s, seconds*.

Due to differences in characteristics of level of injury with residual muscle function, participants were sub-grouped according to the International Standards for Neurological Classification of Spinal Cord Injury (ISNCSCI): motor complete tetraplegia (C1-C8; American Spinal Injury Association impairment scale (AIS) A&B); motor incomplete tetraplegia (C1-C8; AIS C&D); motor complete paraplegia (T1-T12; AIS A&B); and motor incomplete paraplegia (T1-T12; AIS C&D). A mixed model ANOVA was performed to determine significant main and interaction effects for the neurological classifications with respect to time (12, 24, and 36 sessions) and number of steps per session block by mobility test (10MWT, 6MWT, and TUG). *Post hoc* analyses were performed using a paired *t*-test to compare performances of walking assessments from 12 to 36 sessions within the level and completeness sub-groups.

## Results

### Participants

A total of 50 individuals (average age 39 ± 14 years) completed 36 sessions of EAW training. Demographic information for gender, height, weight, duration of injury, level of injury, ISNCSCI classification, and device used are summarized (Table 3).

**Table 3:** Demographic and spinal cord injury characteristics of the total study group.

	**Count (*****N*****) and percent (%)**		**Demographic characteristics**				**SCI characteristics**				
			**Age**	**Height**	**Weight**	**Body mass index**	**Duration of injury**	**Motor complete**		**Motor incomplete**	
								**(AIS A/B)**		**(AIS C/D)**	
**Category**	**N**	***%***	**Years**	**cm**	**kg**	**kg/m**^**2**^	**Years**	***n***	***%***	***n***	***%***
All	50	100	38.68 ± 14.15	174.14 ± 10.33	72.80 ± 13.44	23.94 ± 3.65	4.69 ± 5.18	31	62	19	38
Males	38	76	39.87 ± 14.78	178.00 ± 8.52	76.80 ± 11.67	24.24 ± 3.44	5.37 ± 5.63	26	52	12	24
Females	12	24	34.9 ± 11.68	161.93 ± 4.07	60.14 ± 10.80	22.97 ± 4.25	2.55 ± 2.46	5	10	7	14
Para	36	72	37.44 ± 12.68	173.85 ± 10.08	72.16 ± 13.40	23.83 ± 3.80	4.99 ± 5.78	27	54	9	18
Tetra	14	28	41.86 ± 17.50	174.90 ± 11.31	74.45 ± 13.93	24.22 ± 3.36	3.93 ± 3.22	4	8	10	20
Males-Para	28	56	39.21 ± 13.31	177.53 ± 8.04	74.92 ± 12.53	23.74 ± 3.58	5.77 ± 6.27	22	44	6	12
Males-Tetra	10	20	41.70 ± 19.01	179.32 ± 10.10	82.06 ± 6.88	25.64 ± 2.72	4.25 ± 3.31	4	8	6	12
Female-Para	8	16	31.25 ± 8.01	160.97 ± 3.83	62.48 ± 12.41	24.12 ± 4.75	2.26 ± 2.16	5	10	3	6
Females-Tetra	4	8	42.25 ± 15.59	163.83 ± 4.40	55.45 ± 4.93	20.66 ± 1.74	3.12 ± 3.28	0	0	4	8
DOI > 2 years	26	52	38.15 ± 13.39	174.97 ± 8.60	74.79 ± 12.98	24.37 ± 3.67	7.85 ± 5.55	16	32	10	20
DOI ≤ 2 years	24	48	39.25 ± 15.20	173.25 ± 12.05	70.65 ± 13.87	23.47 ± 3.65	1.28 ± 0.54	15	30	9	18
ReWalk para	27	54	35.63 ± 11.07	174.32 ± 9.77	72.59 ± 13.38	23.88 ± 4.04	5.73 ± 6.43	20	4	7	14
ReWalk tetra	1	2	31	188.96	84.37	23.88	5.00	0	0	1	2
Ekso para	9	18	42.89 ± 16.15	172.44 ± 11.46	70.86 ± 14.16	23.67 ± 3.14	2.79 ± 2.14	7	14	2	4
Ekso tetra	13	26	42.69 ± 17.92	173.89 ± 11.10	73.69 ± 14.19	24.24 ± 3.49	3.85 ± 3.33	4	8	9	18

*Values represent means and (standard deviations); SCI, spinal cord injury; AIS, American Spinal Injury Association Impairment Scale; cm, centimeters; kg, kilograms; m, meters; G1, Group 1; G2, Group 2; Para, paraplegia; and Tetra, tetraplegia; EAW, exoskeletal-assisted walking; UA, usual activities; DOI, duration of SCI; ReWalk or Ekso user for the study as indicated*.

The proportion of males (76%) and females included in this study corresponds with reported proportion of males (about 78%) and females in the United States SCI population (NSCISC, 2019). More individuals with paraplegia participated in this study mainly due to the need of arm and hand function in order to safely use crutches or a walker to maintain balance. Most participants with injury level of T3 or lower used the ReWalk and participants with injury level higher than T3 used the Ekso. However, there were some participants that were thought to be better suited for the other device. This resulted in a total of 28 participants that trained in the ReWalk and 22 that trained with the Ekso (Table 3).

There were no “probably study-related” SAEs, but there were four “possibly study-related” SAEs. There were 49 total study-related AEs which included 39 skin abrasions/bruising, eight musculoskeletal/edema, and two falls. All study-related skin abrasions and musculoskeletal AEs were resolved, and participants continued in study. There were two falls during EAW, but no injuries occurred. Participants had appropriate HR and BP responses throughout the training sessions. RPE's during training ranged from very, very light to very hard (Tapio, 2016). There were no HR or BP-related AEs during EAW.

### Total Steps Results

**Figure 2:**
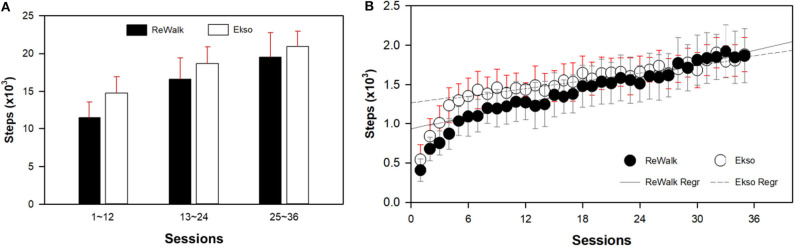
Results of average steps per session block **(A)** and by session **(B)** split by device. Both those using the ReWalk (*r*^2^ = 0.0956, *y* = 27.90x+931.24) and Ekso (*r*^2^ = 0.082, *y* = 16.62x+1267.96) took more steps during later sessions. Since the first 6 sessions were pilot sessions where the participants were introduced to the device, the linear regression models were performed with data from sessions 7 to 36. The Ekso users increased the number of steps per session by 6 to 12 sessions then plateaued, whereas, the ReWalk users initially had less steps per session, but progressively increased by 36 sessions.

There were no order effects for Group 1 (immediate) vs. Group 2 (delayed therapy) for total steps and for any of the walking test results. Descriptive statistics were used to determine mean and standard deviation for the cumulative total number of steps for all sessions. The average number of steps per session by session 36 for all participants (*N* = 50) regardless of the device were 51,065 ± 17,836 and the average steps per session were 1,420 ± 491. The cumulative total number of steps taken across all sessions for all participants split by device is presented in relation to the fastest walking velocity achieved by 36 sessions (Figure 2A). The number of steps taken per session increased overall sessions for both devices. Participants who used the ReWalk took significantly less steps per session during the first 12 sessions than participants who used the Ekso. However, during the last 12 sessions (sessions 25–36) participants who used the ReWalk were able to take more steps per session than those who used the Ekso. Ultimately, participants who used the Ekso took more total overall steps than those who used the ReWalk. The first 6 sessions were pilot sessions where the participants were introduced to the device and had the actual training. The linear regressions of ReWalk (*r*^2^ = 0.0956, *y* = 27.90x + 931.24) and Ekso (*r*^2^ = 0.082, *y* = 16.62x + 1267.96) were performed with steps only on sessions 7–36 (Figure 2B, Table 4).

**Table 4:** Walking test results by devices (ReWalk and Ekso).

**Walking test**	**Unit**	**Session 12**		**Session 24**		**Session 36**	
		**ReWalk**	**Ekso**	**ReWalk**	**Ekso**	**ReWalk**	**Ekso**
10MWT	Mean ± SD (s)	29.51 ± 7.80	50.08 ± 13.59	26.89 ± 7.53	48.37 ± 12.41	25.61 ± 7.89	40.02 ± 12.87
	Min–Max (s)	19.98–46.17	29.90–83.43	17.80–46.60	24.70–70.58	16.58–47.50	21.03–63.68
	Mean ± SD (m/s)	0.36 ± 0.09	0.21 ± 0.06	0.40 ± 0.10	0.22 ± 0.06	0.42 ± 0.11	0.27 ± 0.08
6MWT	Mean ± SD (m)	119.50 ± 32.68	74.78 ± 18.01	136.85 ± 38.50	80.05 ± 20.93	148.44 ± 32.99	96.79 ± 28.94
	Min–Max (m)	59.70–168.40	43.75–112.70	40.70–196.40	46.53–141.80	65.40–206.60	54.60–162.00
	Mean ± SD (m/s)	0.33 ± 0.09	0.21 ± 0.05	0.38 ± 0.11	0.22 ± 0.06	0.41 ± 0.09	0.27 ± 0.08
	FIM 1 to 3 (n, %)	10 (38%)	9 (47%)	8 (30%)	4 (18%)	2 (7%)	2 (9%)
	FIM 4 to 5 (n, %)	14 (54%)	10 (53%)	14 (52%)	18 (82%)	15 (56%)	19 (86%)
	FIM 6 (n, %)	2 (8%)	–	5 (18%)	–	10 (37%)	1 (5%)
TUG	Mean ± SD (s)	67.17 ± 15.25	90.33 ± 18.33	69.69 ± 33.82	81.95 ± 24.11	53.41 ± 11.24	72.22 ± 20.47
	Min–Max (s)	43.72–108.78	64.90–134.37	34.59–155.24	39.05–144.60	35.15–81.50	42.61–103.38
	FIM 1 to 3 (n, %)	12 (52%)	17 (89%)	14 (58%)	12 (54%)	7 (27%)	7 (33%)
	FIM 4 to 5 (n, %)	10 (43%)	2 (11%)	8 (33%)	10 (46%)	12 (46%)	13 (62%)
	FIM 6 (n, %)	1 (5%)	–	2 (9%)	–	7 (27%)	1 (5%)
Total Steps by Sessions	Mean ± SD (#)	11,789 ± 5,421	14,357 ± 5,186	28,788 ± 12,441	32,760 ± 9,892	50,475 ± 19,393	53,685 ± 13,645
	Min–Max (#)	3,620–21,459	4,963–21,516	8,925–47,493	13,163–48,352	21,005–85,125	22,633–74,772
Grouped Total Steps^a^	Mean ± SD (#)	11,789 ± 5,421	14,357 ± 5,186	16,999 ± 7,455	18,403 ± 5,078	209,52 ± 8,246	20,925 ± 4,544
	Min–Max (#)	3,620–21,459	4,963–21,516	5,025–28,820	8,200–28,431	9,338–38,677	9,470–27,767
Steps within Session	Mean ± SD (#)	1,173 ± 594	1,256 ± 345	1,426 ± 593	1,538 ± 430	1,718 ± 731	1,601 ± 349
	Min–Max (#)	210–2,300	423–1,835	427–2,511	389–2,556	143–3,689	745–2,108

*10MWT, ten meter walk test in meters (m); 6MWT, six-minute walk test in seconds (s); TUG, timed up and go in seconds (s); SD, standard deviation; m/s, meters per second; Min, minimum values achieved; Max, maximum value achieved; n, number and %, percent of participants who achieved the criteria; Com, motor complete; Tetra, tetraplegia; Inc, motor incomplete; Para, paraplegia; FDA, Food and Drug Administration; and FIM, Functional Independence Measure. Shaded areas indicate the FIM scores (FIM definitions are reported in Table 5 legend) for 6MWT and TUG walking tests at 12, 24, and 36 sessions. Not all participants had a FIM score recorded during Sessions 12, 24, and 36*.

a *Grouped Total Steps were defined as grouped sessions: 1–12, 13–24, and 25–36*.

### 10 Meter Walk Test (10MWT) Results

At session 12, 92% of the participants performed the 10MWT in ≤ 60 s (≥0.17 m/s). Participants were able to perform the 10MWT with an average of 38.6 ± 14.8 s by 12 sessions. The fastest 10MWT at 12 sessions was 20.0 s and the slowest was 83.4 s. By 36 sessions, 82% of the participants (compared with 62% at session 12) were able to perform the 10MWT in ≤ 40 s (≥0.25 m/s). The average 10MWT across all participants was 36.3 ± 14.6 s by 24 sessions and 32.1 ± 12.6 by 36 sessions. With 36 sessions of EAW training, 17 of 50 participants (34%) fulfilled the FDA 10MWT requirement (≥0.40 m/s) for personal use prescription (Tables 5, 6).

**Table 5:** Number and percent of participants by walking velocity categories.

	**Outcomes**	**Velocity and distance categories**	**12 sessions *n* (%)**	**24 sessions *n* (%)**	**36 sessions *n* (%)**
10MWT	**Primary**	≥0.17 m/s	46 (92%)		
	**Primary**	≥0.25 m/s	31 (62%)		41 (82%)
		Slow: <0.25 m/s	21 (42%)	16 (32%)	10 (20%)
		Medium: ≥0.25 to <0.40 m/s	16 (32%)	18 (36%)	22 (44%)
		Fast: ≥0.40 m/s	13 (26%)	16 (32%)	17 (34%)
	**ReWalk (only) users who met FDA velocity and distance criteria** (10MWT: ≥ 0.40 m/s and 6MWT: ≥110 m)	Total (*n* = 28)	9 (32%)	15 (54%)	15 (54%)
		Com Tetra (*n* = 0)	n/a	n/a	n/a
		Inc Tetra (*n* = 1)	1 (100%)	1 (100%)	1 (100%)
		Com Para (*n* = 20)	6 (30%)	11 (55%)	11 (55%)
		Inc Para (*n* = 7)	2 (29%)	3 (43%)	3 (43%)
6MWT	**Primary**	≥50 m	48 (96%)		
	**Primary**	≥80 m	35 (70%)		41 (82%)
		Slow: <80 m	16 (32%)	14 (28%)	8 (16%)
		Medium: ≥80 to <110 m	18 (36%)	12 (24%)	8 (16%)
		Fast: ≥110 m	16 (32%)	24 (48%)	33 (66%)
		FIM ≥4	26 (52%)	37 (74%)	45 (90%)
TUG	**Primary**	≤ 120 s	48 (96%)		
	**Primary**	≤ 90 s	36 (72%)		45 (90%)
		Slow: ≥120 s	1 (2%)	5 (10%)	0 (0%)
		Medium: ≥90 to <120 s	10 (20%)	5 (10%)	5 (10%)
		Fast: <90 s	36 (72%)	38 (76%)	43 (86%)
		FIM ≥4	13 (26%)	20 (40%)	33 (66%)

*10MWT, ten meter walk test in meters (m); 6MWT, six-minute walk test in seconds (s); TUG, timed up and go in seconds (s); SD, standard deviation; m/s, meters per second; Min, minimum values achieved; Max, maximum value achieved; n, number and %, percent of participants who achieved the criteria; Com, motor complete; Tetra, tetraplegia; Inc, motor incomplete; Para, paraplegia; and FDA, Food and Drug Administration. slow, medium, and fast velocity sub-groups. The velocity sub-groups were defined post hoc after the review of data*.

*Shaded areas indicate the Primary outcome results for each of the walking tests at 12 and 36 sessions. The FDA criteria was applied only to the ReWalk users because the Ekso is not indicated for personal use*.

*Modified Functional Independence Measurement (FIM) Scoring Key:*.

*1, Total Assist (performs <25% of task); 2, Maximal Assist (performs 25 to 49% of task); 3, Moderate Assist (performs 50–74% of task); 4, Minimal Assist (performs 75% or more of task); 5, Supervision (cuing, coaxing, prompting); 6, Modified Independence (no assistance, user may require extra time); 7, Complete Independence (timely, safely, no assistance, no devices), albeit, not applicable in this study*.

**Table 6:** Walking test assessment results.

**Sessions**		**12**	**24**	**36**
10MWT	Mean ± SD (s)	38.56 ± 14.80	36.34 ± 14.60	32.08 ± 12.59 ^¥^^T^
	Mean ± SD (m/s)	0.30 ± 0.11	0.32 ± 0.12	0.36 ± 0.12 ^¥^^T^
	Min–Max (s)	20.0–83.4	17.8–70.6	16.6–63.7
6MWT	Mean ± SD (s)	99.83 ± 35.07	111.86 ± 42.61^*^	125.25 ± 40.37 ^¥^°
	Mean ± SD (m/s)	0.28 ± 0.10	0.31 ± 0.12^*^	0.35 ± 0.11 ^¥^°
	Min–Max (s)	43.8–168.4	40.7–196.4	54.6–206.6
TUG	Mean ± SD (s)	78.01 ± 20.28	75.31 ± 30.10	62.03 ± 18.55 ^¥^°
	Min–Max (s)	43.72–134.37	34.59–155.24	35.15–103.38

*SD, standard deviation; m, meters; WT, walk test; min, minutes; TUG, timed up and go; sec, seconds; and m/s, meters per second. Sessions 12 vs. 24*:

* *p < 0.0001; Sessions 12 vs. 36*:

¥ *p < 0.0001; and Sessions 24 vs. 36*:

T *p = 0.0008*,

° *p < 0.0001*.

### Six Minute Walk (6MWT) Test Results

Thirty-five participants (70%) were able to walk a distance ≥80 meters for the 6MWT by 12 sessions. Twenty-six participants (52%) achieved successful EAW training with or without minimal assistance by 12 sessions. Forty-eight participants (96%) were able to walk more than 50 meters (≥0.14 m/s) in the 6MWT and the average 6MWT was 99.8 ± 35.1 m at 12 sessions. By 24 sessions, about half of the participants (24 participants, 48%) were able to meet FDA requirements for the 6MWT (≥110 m). By 36 sessions, 41 (82%) participants accomplished a 6MWT of ≥80 m (≥0.22 m/s). The average 6MWT was 111.9 ± 42.6 m by 24 sessions and 125.3 ± 40.4 m by 36 sessions. At 36 sessions of EAW training, 33 of 50 participants (66%) fulfilled the FDA requirement for the 6MWT (110 m) for personal use prescription (Tables 5, 6).

### Timed Up and Go (TUG) Test Results

At session 12, 46 participants (92%) performed the TUG in 120 s and 36 participants (72%) performed the TUG in <90 s. By Session 36, 84% of the participants were able to perform the TUG in <90 s (Tables 5, 6).

### Combined Walking Test Result Reporting

The number and percent of participants who were categorized by slow, medium, and fast walkers, their progression into the more skillful category over the three timepoints (sessions 12, 24, and 36) and number of participants who met FDA velocity criteria, stratified by level of injury are presented (Table 5). With 36 sessions of EAW training, 15 of 50 participants (30%) who used the ReWalk succeeded in achieving both of FDA speed requirements for personal use prescription (10MWT within 25 s or ≥0.40 m/s and 6MWT ≥110 m or ≥0.31 m/s). Those fifteen participants met the FDA requirement by 24 sessions (Table 5).

The overall performance results from the different walking assessments at the three time points, the change in performance with additional training sessions and the range of speeds achieved, respectively, are presented (Table 6). A repeated measures ANOVA with a Greenhouse-Geisser correction determined that the mean of 10MWT, 6MWT, and TUG differed statistically between time points (10MWT: (*F*_(1.841, 88.372)_ = 13.921, *p* < 0.0005), 6MWT (*F*_(1.849, 88.734)_ = 34.830, *p* < 0.0005), and TUG (*F*_(1.597, 68.665)_ = 13.749, *p* < 0.0005)). Paired-sample *t*-tests were conducted to compare the performance of tasks with the number of sessions. There were no significant differences in the 10MWT and the TUG from 12 to 24 sessions. However, there were significant differences in all mobility assessments, 10MWT, 6MWT, and TUG from 24 to 36 sessions. There were also significant differences from 12 to 36 sessions. The mean values for the 10MWT (s), 6MWT (m), and TUG (s) walking assessments are presented (Table 6). The average results of all participants' walking velocities and distances from 12 to 36 sessions were significantly improved (Table 6).

Using the walking velocity, participants were divided into three sub-groups: slow, medium, and fast. The results of the TUG showed most of participants (82%) falling into the medium and fast velocity sub-groups at session 12. This improved with further training, as 86% of participants fell in the fast category at session 36. It was hypothesized that 20% of participants at 12 sessions and 60% of participants at 36 sessions would be able to perform the TUG in ≤ 90 s. However, more than two thirds of participants (72%) accomplished TUG criterion at session 12 and 90% of participants did at session 36 in ≤ 90 s. Using the walking velocity from the 10MWT, the average TUG was calculated for the three velocity sub-groups and presented (Table 7).

**Table 7:** Average TUG by velocity sub-groups of 10MWT speed.

			**TUG**					
			**Session 12**		**Session 24**		**Session 36**	
			***N***	**Mean ± SD**	***N***	**Mean ± SD**	**N**	**Mean ± SD**
10 MWT	≤0.25 m/s	Slow	20	89.61 ± 19.75	16	87.84 ± 24.43	10	83.25 ± 16.76
	0.25 ≤ Speed ≤ 0.4	Medium	15	74.93 ± 18.40	17	76.24 ± 32.01	23	59.48 ± 16.43
	≥0.4 m/s	Fast	12	62.53 ± 9.84	15	60.88 ± 28.77	15	51.80 ± 10.32

*10MWT, ten-meter walk test in meters; TUG, timed up and go in seconds; SD, standard deviation; m/s, meters per second*.

*TUG was defined/calculated by the velocity sub-groups of 10MWT at 12, 24, and 36 sessions*.

### Comparison Between Devices

**Figure 3:**
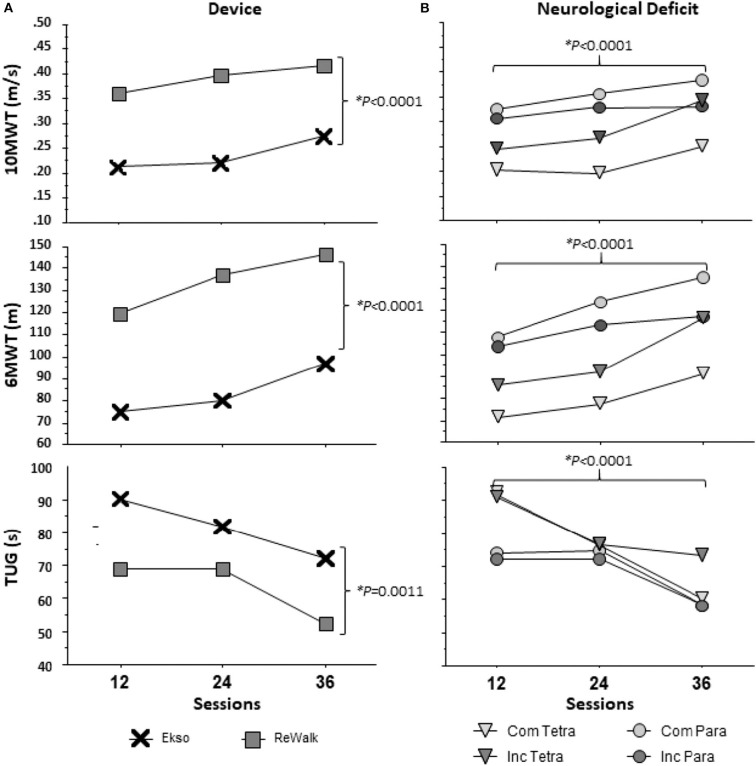
Results of Walking Tests across 12, 24, and 36 sessions by **(A)** device and **(B)** neurological deficit. Neurological deficit: Com Tetra (Motor Complete Tetraplegia); Inc Tetra (Motor Incomplete Tetraplegia); Com Para (Motor Complete Paraplegia); and Inc Para (Motor Incomplete Paraplegia). The main effects for neurological deficit (ANOVA: 10MWT (*F*_(3, 46)_ = 2.568, *p* = 0.658), 6MWT (*F*_(3, 46)_ = 2.267, *p* = 0.0933), TUG (*F*_(3, 46)_ = 0.946, *p* = 0.4263)) were not significantly different, but the main effects for sessions and device (10MWT: *p* < 0.0001, 6MWT: *p* < 0.0001, TUG-12: *p* = 0.0006, TUG-24: *p* = 0.1299, TUG-36: *p* < 0.0001) were statistically significant for each walk test as shown.

Due to the different characteristics between the ReWalk and Ekso, the results from the 10MWT, 6MWT, and TUG were significantly different by device. By session 36, the fastest participant in the ReWalk performed the 10MWT in 16.6 s and slowest in 47.5 s, whereas in the Ekso the fastest was 21.0 s and the slowest was 63.7 s (Table 4, Figure 3A).

### Effect of Neurological Deficit

Change in walking test performance was independent of neurological deficit. As mentioned previously, participants were divided by four neurological deficit sub-groups: motor complete tetraplegia (*n* = 4); motor incomplete tetraplegia (*n* = 10); motor complete paraplegia (*n* = 27); and motor incomplete paraplegia (*n* = 9). There were no significant differences between groups in terms of improvements from 12 to 36 sessions on the 10MWT [one-way ANOVA (*F*_(3, 45)_ = 2.555, *p* = 0.067)], 6MWT[one-way ANOVA (*F*_(3, 45)_ = 1.150, *p* = 0.339)], and TUG [one-way ANOVA (*F*_(3, 41)_ = 1.115, *p* = 0.354)]. Within level and completeness sub-groups, paired *t*-tests were used to compare the performance of tasks from 12 to 36 sessions. Overall, those with complete tetraplegia walked shorter distances in the 6MWT and took more time for the 10MWT and TUG at session 12. Participants with complete paraplegia performed the best among the sub-groups for 10MWT and 6MWT at session 12. From sessions 12 to 36, those with complete tetraplegia demonstrated no significant change in the 10MWT and 6MWT, however, there was significant improvement on TUG (*p* = 0.019). All walking assessments were significantly improved from 12 to 36 sessions in the sub-groups of incomplete tetraplegia (10MWT: *p* = 0.020, 6MWT: *p* = 0.011, TUG: 0.046) and complete paraplegia (10MWT: *p* = 0.001, 6MWT: *p* < 0.000, TUG: *p* = 0.002). Both those with incomplete tetraplegia and complete paraplegia demonstrated improvement in the TUG (*p* = 0.015). Each sub-group's results of the walking tests are reported at 12, 24, and 36 sessions (Figure 3B).

**Figure 4:**
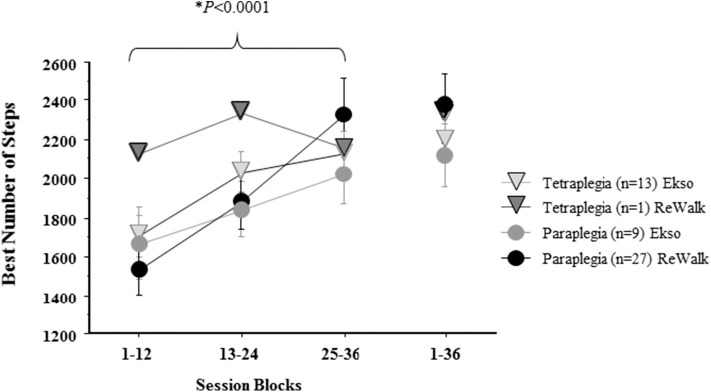
The average best number of total steps in a session block by level of SCI by device. The mean ± standard error of the best number of total steps/sessions achieved during each 12-session block by Tetra (Tetraplegia), Para (Paraplegia) and by device (ReWalk, Ekso) are reported. The overall best number of steps in a single session is reported for 1–36. The number of steps significantly increased by session block, but no significant effects were found for combination of Tetra/Para and Device.

Using the average of the highest achieved number of steps per session block (between 1 and 12, 13 and 24, 25 and 36) split by tetraplegia/paraplegia and device, a repeated measures ANOVA with a Greenhouse-Geisser correction determined that the number of steps differed statistically between session blocks (*F*_(1.336, 66.477)_ = 39.868, *p* < 0.0001). However, there were no significant differences among sub-groups (tetraplegia/paraplegia and device) in terms of number of steps from session block 1–12 [one-way ANOVA (*F*_(3, 46)_ = 0.507, *p* = 0.679)], session block 13-24 [one-way ANOVA (*F*_(3, 46)_ = 0.364, *p* = 0.779)], and session block 25-36 [one-way ANOVA (*F*_(3, 46)_ = 0.437, *p* = 0.728)] (Figure 4).

**Figure 5:**
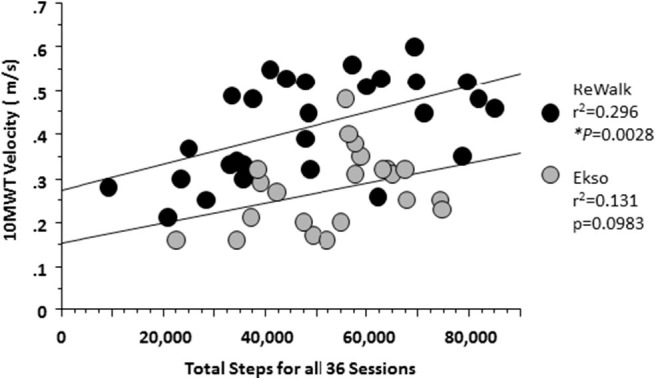
Relationship between the total steps and 10MWT velocity at 36 session by Device. At 36 sessions, participants using either device showed that with more steps taken there was an associated increase in 10MWT velocity. A significant relationship was noted for those who used the ReWalk (*r*^2^ = 0.296, *p* = 0.0028) and a trend for those who used the Ekso (*r*^2^ = 0.131; *p* = 0.0983).

Regardless of device there was a positive relationship between the total cumulative number of steps taken during the 36 sessions and the maximum 10MWT velocity achieved. ReWalk users had a stronger relationship than those who used the Ekso (*p* = 0.0028 vs. *p* = 0.093, respectively) (Figure 5).

## Discussion

More than half of the participants succeeded in achieving hypothesized milestones of ≤ 40 s for the 10MWT, ≥80 m for the 6MWT and ≤ 90 s for TUG using EAW by session 12 and more than 80% of the participants achieved them by session 36. The rate of improvement in the walking tests was unrelated to the level, completeness, or duration of SCI. These findings indicate that improving the skill level of using these devices as measured by walking velocity and distance is achievable across a broad spectrum of SCI level and completeness. Among neurological sub-groups, there were no significant differences in improvements on walking assessments. Participants with complete paraplegia performed better than participants with complete tetraplegia for all walking assessments (10MWT, 6MWT, and TUG) during all time points, but there were no differences between incomplete tetraplegia and incomplete paraplegia by session 36. This was expected, as those with lower levels of injury retain more residual motor control over their body, allowing them to control thoracic movements in the device, and translating into a better ability to perform exoskeletal ambulation. All participants in the complete tetraplegia sub-group used the Ekso for this study. Participants with lower level injury more often were placed in the ReWalk group. The study was not designed to determine differences in the mobility test outcomes between the Ekso and ReWalk groups. However, the faster walking velocities in the ReWalk may have been due to differences in level and completeness of injury as well as differences between the devices' engineering characteristics.

It may not be practical for clinicians to provide 36 sessions of EAW training due to limitations in payment for physical therapy visits, especially for personal prescription (i.e., use in the home and community). However, participants who met FDA criteria (10MWT: speed ≥0.40 m/s and 6MWT: distance ≥110 m) mastered weight shifting while standing and clearing the foot for stepping within 24 sessions. Nine participants achieved this by session 12, and 15 achieved it by session 24, and continued to meet these criteria at session 36. Future investigations focused on the different characteristics of the participants that would eventually obtain the skill needed to pass the FDA criteria should be explored. This could then be used to formulate a basic screening test to identify participants most likely to achieve the skills needed to pass the FDA criteria. Although the number of covered physical therapy visits vary depending on insurance, in general there is a cap at about 20 visits for Medicare and Medicaid patients. Our data suggest that the “sweet spot” for achieving the FDA criteria for most individuals falls between 12 and 24 visits, and is in alignment with current Center for Medicare and Medicaid Services (CMS) reimbursement guidelines.

There was high variability in the total number of steps taken in both devices. This may be accounted for by participant motivation, confidence in the device, stamina, and/or total time attended per session. All participants walked more steps with the progression of sessions. On average, Ekso users took more total cumulative steps than ReWalk users. However, the average number of steps during later sessions and within session 36 for ReWalk users were higher than those for Ekso users. Overall, the ReWalk users were faster than the Ekso users. While the participants who used the ReWalk were generally able to walk faster, this device was limited to those individuals with a greater amount of trunk stability (based on ISNCSCI level) and strong enough hand grasp to use crutches without any type of assistance. Greater trunk stability and strength likely improved balance and made the performance of weight shifting easier. Our findings suggest that the Ekso is easier to learn to use than the ReWalk initially, but once learned, the ReWalk user has more flexibility to control velocity and achieve faster walking speeds. The Ekso users increased the number of steps per session early in training with many reaching near their peak steps by session 12, and then they plateaued. On the other hand, the ReWalk users initially had less steps per session, but progressively increased by 36 sessions. This is likely a design feature, since the Ekso can provide more hip and knee flexion assistance than the ReWalk, making it easier to learn to use the device. Ekso users were able to achieve higher number of steps early and continue to steadily increase stepping throughout session progression. ReWalk use required more trunk control over the device to successfully take steps and has a higher initial learning curve to achieve proper posture, weight shifting and stepping for many participants.

Even with the limitation of the device characteristics, there were two Ekso users in the sub-group of incomplete tetraplegia who met the fast walking velocity criteria. While these two Ekso users were daily power wheelchair users and had cervical levels of SCI, they were functionally able to take a few steps without the exoskeleton but with an assistive device such as a walker and with physical assistance from another person. Remarkably, these two Ekso users met all hypothesized criteria of nominal velocity and distance by session 12. Although the ReWalk requires trunk stability and strong enough hand grip to use the crutches, in the incomplete tetraplegia sub-group, there was one person who used the ReWalk and met the fast walking criteria (i.e., the FDA personal use criteria). In contrast, only three of the nine participants with low paraplegia made the FDA criteria in the sub-group of incomplete paraplegia who used the ReWalk. One participant with low paraplegia who used the ReWalk was partially able to meet FDA criteria (6MWT: distance ≥110 m), although the person had performed all hypothesized study criteria of nominal velocity and distance by session 12.

In summary, most participants who were unable to meet the fast walking velocity criteria were individuals with high level paraplegia or were Ekso users. Unexpectedly, three participants with tetraplegia achieved the FDA criteria even with the severity of their neurological deficit. Based on these results, selection of the proper device should not solely be defined by neurological deficit, but other factors such as user preference, comfort and fit, and skill ability as determined by a short trial of devices. Although it is recognized that the number of sessions during training may be limited to policies of third-party payers or government insurance coverage, when possible, the duration and number of individualized EAW mobility training sessions should be determined by participants' stamina, motivation, residual function, and strength, and not just the level or completeness of the SCI.

To minimize trainer support and help the user gain reasonable independence, it is important to establish appropriate goal setting and time management for EAW mobility training. When personal prescription is the goal, an efficient EAW mobility skills training should be implemented. Following guidelines already established by our group, an effective exoskeleton training program necessitates all components of appropriate candidate selection, proper fitting of the device, a steady skill progression plan, and provision of participant assistance on areas of the body with intact sensation (Asselin et al., 2016). As was the case in a previous report, we used these guidelines for this study. For the effective training program, sufficient education of the elements of EAW must be included. Upon completion of a training program, the user should be able to identify the safe environments for device use and operate the device in simulated or actual use environments representative of indicated environments and use^5^ One of the most important elements is using the devices in actual environments such as noisy or crowded hallways, door navigation, and in spaces where turning is required. The EAW walking tests have been previously reported as reliable for testing achievements in mobility during the walking sessions and were accurate predictors of functional independence in the home and community (Louie et al., 2015). Our data confirm the reliability of these tests. There are no specific FDA criteria for the TUG although it is important to measure. The TUG is an essential skill because users must be proficient at standing up, walking, turning and sitting down. Our hypothesized minimal criteria for TUG success were ≤ 120 s by session 12 and ≤ 90 s by session 36 sessions. These criteria were easy to achieve compared to 10MWT and 6MWT. According to our average TUG data set of 10MWT, the TUG criterion to ≤ 75 s by session 12 and ≤ 60 s by session 36 sessions would be more discriminative. Thus, TUG ≤ 60 s would be suggested as a benchmark for skill proficiency. This more stringent TUG criterion could be used to support a skill level needed to take the device home, as it encompasses additional skills and is not solely focused on walking speed.

## Conclusions

EAW training was demonstrated to be safe, feasible, and effective within a 36-session training timeline. Most participants improved their walking velocity and distances with the progression of sessions. The observed combination of how the Ekso triggers stepping and higher step clearance allowed participants to walk more successfully during the earlier sessions. Whereas, ReWalk users usually needed more sessions to learn appropriate weight shifting to better trigger stepping and to clear the foot during the swing phase, but once they learned this skill, they walked at faster velocities. More than half of the ReWalk users were able to meet FDA velocity criteria for personal prescription. Our data suggests that clinical programs can expect success rates of 58% by 12 sessions, 68% by 24 sessions and 78% by 36 session to achieve walking velocity medium and fast milestones of ≥0.25 m/s and ≥0.40 m/s, respectively, regardless of level and completeness of injury or device used. The results from this study provide guidelines for estimating the potential of individuals with SCI to achieve proficient and safe EAW mobility skills for institutional and personal use of these devices.

## Data Availability Statement

All datasets generated for this study are included in the article/supplementary material.

## Ethics Statement

The studies involving human participants were reviewed and approved by Institutional Review Boards (IRB) of James J. Peters VA Medical Center (JJPVAMC), Bronx, NY, Kessler Institute for Rehabilitation/Kessler Foundation (KIR/KF), University of Maryland, Baltimore IRB for the University of Maryland Rehabilitation and Orthopaedic Institute (UM Rehab and Ortho, Baltimore, MD), and Department of Defense Congressionally Directed Medical Research Program (DOD CDMRP) IRB. The patients/participants provided their written informed consent to participate in this study.

## Author Contributions

AS, PG, GF, and PA designed the study. AS, PG, GF, EH, PA, SKn, SKo, WS, and SW contributed to the implementation of the research, to the analysis of the results, and to the writing of the manuscript. All authors contributed to the article and approved the submitted version.

## Conflict of Interest

The authors declare that the research was conducted in the absence of any commercial or financial relationships that could be construed as a potential conflict of interest.

## Footnotes

^1^ReWalk. Available online at: https://rewalk.com/.

^2^Ekso Bionics. Available online at: https://eksobionics.com/eksohealth/.

^3^Perceived Exertion (Borg Rating of Perceived Exertion Scale): Centers for Disease Control and Prevention. Available online at: https://www.cdc.gov/physicalactivity/basics/measuring/exertion.htm.

^4^10 Meter Walking Test: Physiopedia. Available online at: https://www.physio-pedia.com/10_Metre_Walk_Test.

^5^Electronic Code of Federal Regulations. Available online at: https://www.ecfr.gov/cgi-bin/text-idx?SID=9b627fd1822b8fcd87ad660db602de1candmc=trueandnode=pt21.8.890andrgn=div5%E2%80%9D%5Cl%E2%80%9Cse21.8.890_13480#se21.8.890_13480.
